# Genetic manipulation of porcine deltacoronavirus reveals insights into NS6 and NS7 functions: a novel strategy for vaccine design

**DOI:** 10.1080/22221751.2019.1701391

**Published:** 2019-12-20

**Authors:** Mengjia Zhang, Wan Li, Peng Zhou, Dejian Liu, Rui Luo, Anan Jongkaewwattana, Qigai He

**Affiliations:** a State Key Laboratory of Agricultural Microbiology, College of Veterinary Medicine, Huazhong Agricultural University, Wuhan, People’s Republic of China; b Key Laboratory of Preventive Veterinary Medicine in Hubei Province, the Cooperative Innovation Center for Sustainable Pig Production, Wuhan, People’s Republic of China; c Virology and Cell Technology Laboratory, National Center for Genetic Engineering and Biotechnology (BIOTEC), National Science and Technology Development Agency (NSTDA), Pathum Thani, Thailand

**Keywords:** PDCoV, infectous clone, accessory protein, pathogenesis, vaccine

## Abstract

Porcine deltacoronavirus (PDCoV) is an emerging swine coronavirus that causes severe diarrhea, resulting in high mortality in neonatal piglets. Despite widespread outbreaks in many countries, no effective PDCoV vaccines are currently available. Here, we generated, for the first time, a full-length infectious cDNA clone of PDCoV. We further manipulated the infectious clone by replacing the NS6 gene with a green fluorescent protein (GFP) to generate rPDCoV-ΔNS6-GFP; likewise, rPDCoV-ΔNS7 was constructed by removing the ATG start codons of the NS7 gene. Growth kinetics studies suggest that rPDCoV-ΔNS7 could replicate similarly to that of the wild-type PDCoV, whereas rPDCoV-ΔNS6-GFP exhibited a substantial reduction of viral titer *in vitro* and *in vivo*. Piglets inoculated with rPDCoV-ΔNS7 or wild-type PDCoV showed similar diarrheic scores and pathological injury. In contrast, rPDCoV-ΔNS6-GFP-infected piglets did not show any clinical signs, indicating that the NS6 protein is an important virulence factor of PDCoV and that the NS6-deficient mutant virus might be a promising live-attenuated vaccine candidate. Taken together, the reverse genetics platform described here not only provides more insights into the role of PDCoV accessory proteins in viral replication and pathogenesis, but also allows the development of novel vaccines against PDCoV infection.

## Introduction

Coronaviruses (CoVs) have been recognized as emerging pathogens that cause significant morbidity and mortality, leading to major economic losses in both humans and animals [1]. They are currently classified into four genera, the Alpha-, Beta-, Gamma-, and Deltacoronavirus, identified in diverse animal species including humans [2]. With its origin still unknown, porcine deltacoronavirus (PDCoV) is a recently emerged deltacoronavirus in swine. It particularly targets neonatal piglets, causing acute diarrhea, vomiting, dehydration and high mortality [3–7]. In 2012, PDCoV was first identified in swine herds in Hong Kong and was subsequently reported as an enteric pathogen causing diarrhea in the United States in 2014 [2,3,7–11]. Shortly thereafter, PDCoV has been reported in South Korea, mainland China and Thailand [4,12,13]. Currently, PDCoV, together with other enteric viruses including porcine epidemic diarrhea virus (PEDV), rotavirus (RV) and transmissible gastroenteritis virus (TGEV), is causing enormous economic losses in the swine industry worldwide [9,14].

PDCoV is a positive-sense single-stranded RNA virus with its genomic RNA approximately 25 kb in length, the smallest among the known coronaviruses [15]. The genome organization is similar to those of other reported delta-CoVs with the typical gene order of: 5′ UTR-ORF1a/1b-S-E-M-NS6-N-NS7-3′ UTR [3,8,12]. ORF1a and ORF1b encode two large replicase precursor polyproteins, pp1a and pp1ab, with the expression of the latter involving a −1 ribosomal frameshift mechanism [16]. According to studies on other CoVs, the replicase pp1a and pp1ab are generally cleaved by virus-encoded proteases into 15 non-structural proteins involved in viral transcription and replication. PDCoV encodes the traditional coronavirus structural proteins, the spike glycoprotein (S), the envelope protein (E), the membrane glycoprotein (M), and the nucleocapsid protein (N). Although general characteristics of the coronaviral proteins and their roles in viral replication have been clarified, the detailed functions and roles of the proteins encoded by PDCoV in host cells and animals are still largely unknown.

The CoV accessory proteins (also known as group- or virus-specific proteins) are strikingly different among CoVs. Each coronavirus contains varying numbers of accessory genes interspaced between and within the viral structural protein genes [17]. Alpha-CoVs including human coronavirus (HCoV)-NL63 and PEDV encode a single accessory protein between the spike and envelope genes. However, other alpha-CoVs such as human coronavirus 229E (HCoV-229E), TGEV and feline infectious peritonitis virus (FIPV) possess two, three and five accessory proteins, respectively [18–21]. For beta-CoVs, mouse hepatitis virus (MHV), HCoV-OC43, and Middle East respiratory syndrome coronavirus (MERS-CoV), each has five accessory proteins, while at least eight accessory proteins have been identified in severe acute respiratory syndrome-associated coronavirus (SARS-CoV) [22]. Infectious bronchitis virus (IBV), a representative gamma-CoV, has four accessory proteins [23].

Although CoVs’ accessory proteins have been generally regarded as dispensable for viral replication *in vitro* [24–26], several of them are known to contribute to viral pathogenesis *in vivo*. For example, replacement the ORF3 with the red fluorescent protein (RFP) gene in PEDV resulted in the partial attenuation of the virus in piglets [27]. Deletion of the ORF 6 in SARS-CoV also led to a significant attenuation in animal models [24]. Similarly, MHV and FIPV mutants bearing defective accessory protein genes were also dramatically attenuated when inoculated into their natural hosts [17,28]. Recently, PDCoV NS6, which is located between the M and N, and NS7, which is occupied within the N gene in an alternative ORF, were identified as the accessory proteins [29]. Moreover, NS7a, a 100-amino-acid-polypeptide identical to the C-terminus of NS7, has also been identified as a novel accessory protein [30]. To the best of our knowledge, no previous reports have addressed the role of PDCoV proteins, particularly the accessory proteins, in viral replication and pathogenicity.

In this study, we successfully generated the full-length infectious cDNA clone of a virulent Chinese PDCoV strain, CHN-HG-2017 [5]. The infectious clone was further manipulated to construct recombinant viruses harbouring defective accessory genes NS6 and NS7. The PDCoV devoid of NS6, but not of NS7, showed a reduction in virus replication in cell cultures and is highly attenuated in neonatal piglets. This study thus underscores the importance of the reverse genetics platform for PDCoV that not only offers the opportunities to gain further insights into PDCoV virulence and pathogenesis, but also provides novel strategies for live-attenuated vaccine development.

## Results

### Design of the full-length cDNA clone of PDCoV

**Figure 1.**
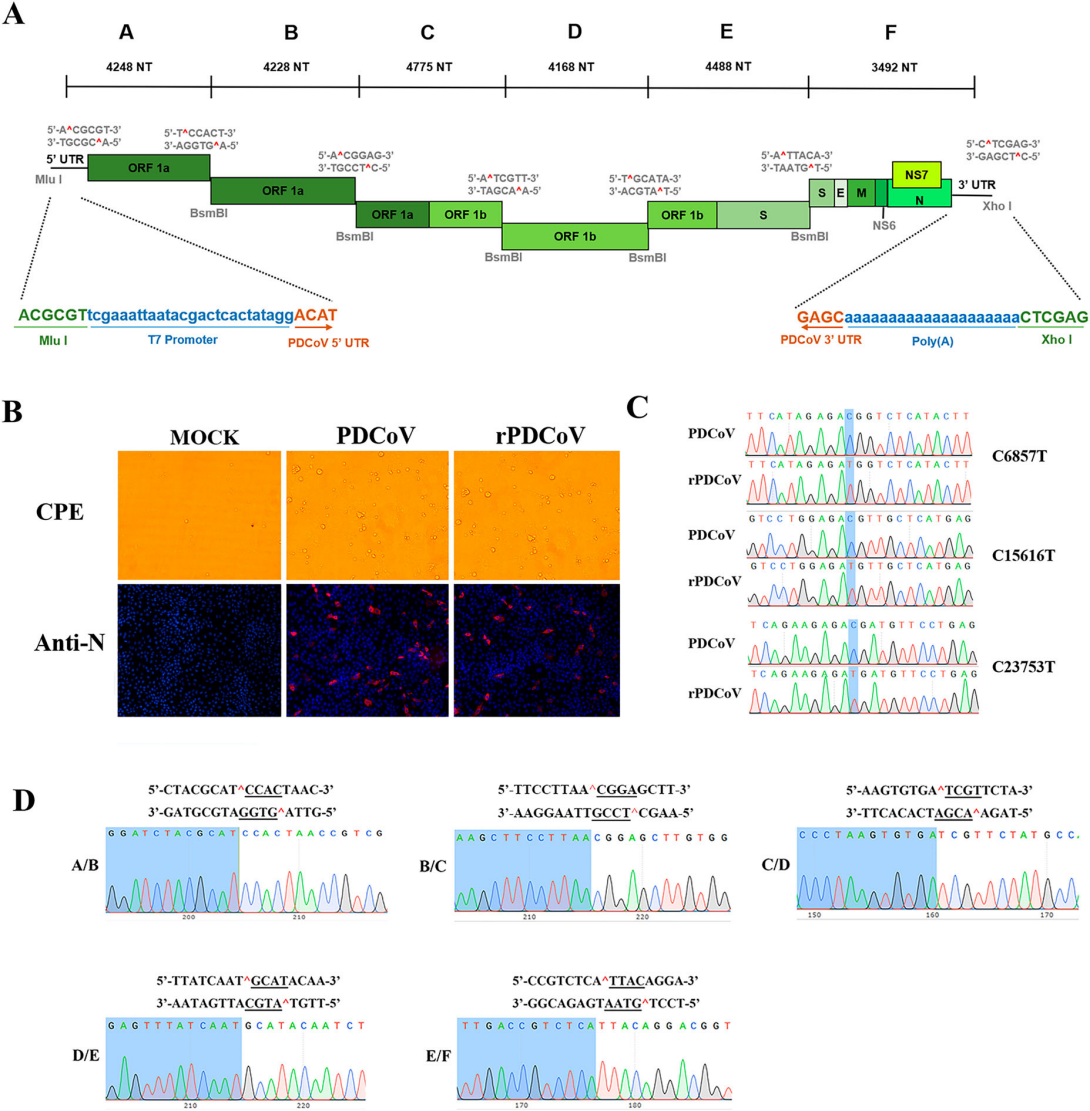
Assembly of a full-length PDCoV cDNA clone and the recovery of rPDCoV. (A) The organization of PDCoV strain CHN-HG-2017 genome and the full-length genome was divided into six contiguous cDNAs designated PDCoV A–F. Restriction sites flanking each fragment are noted. (B) PDCoV-, rPDCoV-infected, or mock-infected LLC-PK1 cells were detected by IFA at 18 h post infection (hpi) using monoclonal antibodies against PDCoV N protein. (C) Three BsmBI restriction sites were removed from rPDCoV, as indicated by C-T changes in blue. (D) Connection between fragments identification by sequencing genome sequences of rPDCoV. Underlined sequences corresponding to the different asymmetric overhangs between each fragment.

To construct the infectious clone of PDCoV, the full-length genome of a virulent PDCoV strain CHN-HG-2017 was sequenced (GenBank: MF095123.1) and the complete viral genome was cloned as six contiguous fragments (A–F) that could be systematically linked by unique BsmBI restriction sites at nucleotide positions 4248, 8476, 13251, 17415, 21907, respectively (Figure 1A). Notably, BsmBI is a type IIS restriction endonuclease that cleaves asymmetric DNA sequences (5′-CGTCTCN↓NNNN-3′) but leaves 4-nucleotide variable overhangs. Thus, these unique overhangs between each fragment allow for the systematic, directional, and efficient assembly of each fragment into the full-length genomic cDNA of PDCoV by *in vitro* ligation. In addition, three naturally occurring BsmBI restriction sites, separately located in fragment B, D, F, were removed by C-T changes at positions 6857, 15616, 23753, respectively. Notably, changes in BsmBI cutting sites gave rise to silent mutations, thereby not affecting protein products. The A fragment contains a T7 promoter, whereas the F fragment terminates in 20 A residues (Figure 1A), allowing the synthesized RNA transcripts with capped and polyadenylated structures as products of *in vitro* transcription.

### Recovery of infectious rPDCoV from the cDNA clone

The full-length PDCoV cDNA was used as a template for *in vitro* transcription with the T7 RNA polymerase. Since the N gene transcripts were found to enhance the infectivity of TGEV, MHV, and SARS-CoV full-length transcripts [31], PDCoV full-length transcripts were mixed with capped PDCoV-N gene transcripts and co-electroporated into LLC-PK1 cells. Within 48–72 h post-transfection, clear cytopathic effects were observed, and the recombinant-virus mRNA could be detected by RT-PCR within transfected cultures, but not in mock-infected cells (data not shown). Following three rounds of plaque purification, the infectivity of rPDCoV in LLC-PK1 cells was confirmed by indirect immunofluorescence assays (IFA) using nucleocapsid (N) protein-specific antibodies (Figure 1B). To examine the genomic identity of the rPDCoV, the genomic RNA of two plaque-purified clones were subject to nucleotide sequencing. As shown in Figure 1C and D, the entire genome sequences of rescued viruses were identical to the cDNA clone, including the genetic markers at position 6857, 15616, 23753 and the junctions between six fragments, thereby suggesting that the rPDCoV was successfully rescued in LLC-PK1 cells.

### Recovery of infectious rPDCoVs bearing disrupted NS6 or NS7 gene

**Figure 2.**
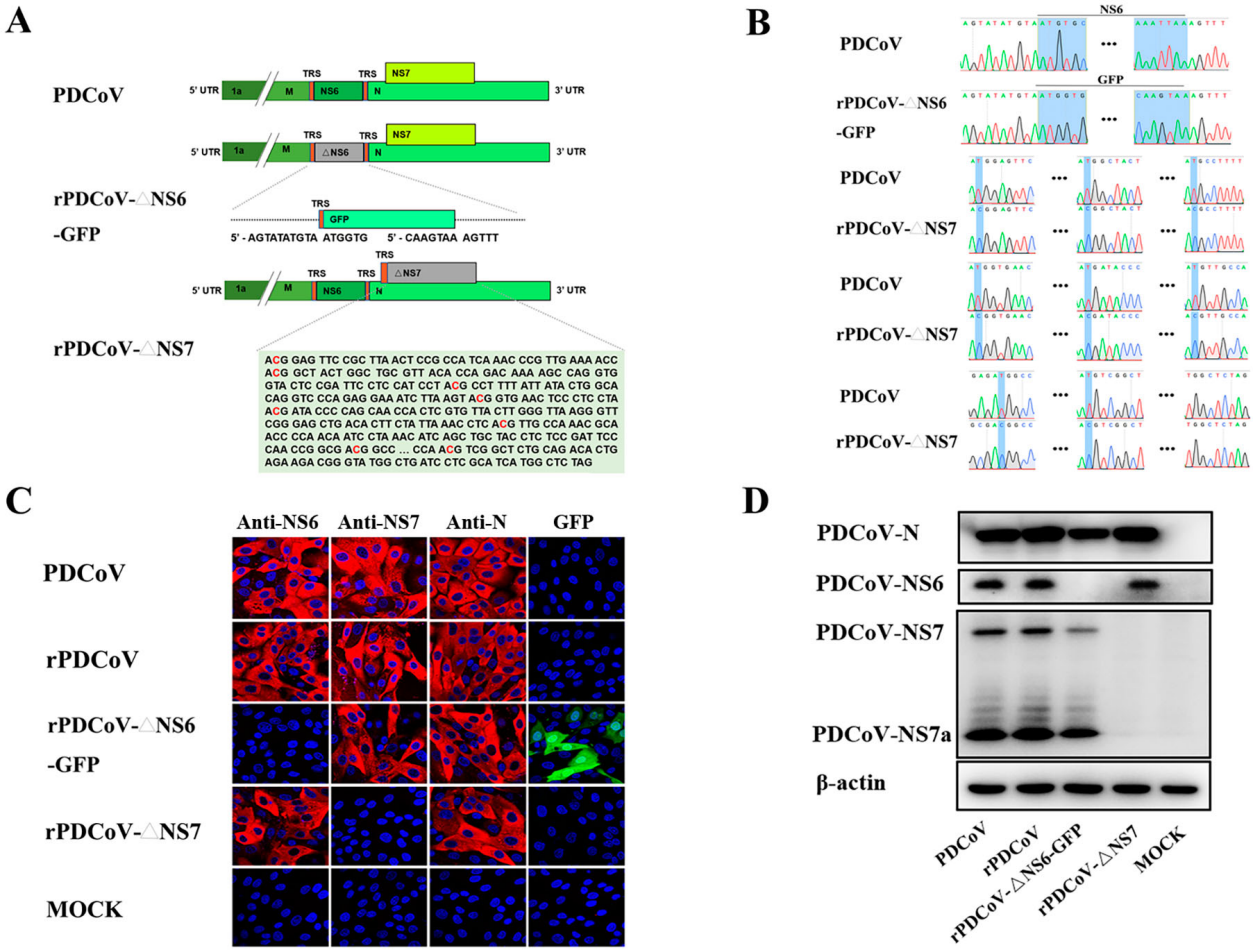
Rescue of rPDCoV-ΔNS6-GFP and rPDCoV-ΔNS7. (A) Schematic representation of recombinant PDCoV cDNA clone with NS6 or NS7 deletion. To generate NS6-deleted virus, NS6 in the PDCoV F fragment was replaced with a green fluorescent protein (GFP). To construct NS7-deleted virus, initiation codons ATG and the following seven downstream ATGs of the NS7 gene were changed to ACGs to construct rPDCoV without NS7 expression. (B) Mutation identification by sequencing genome sequences of rPDCoV-ΔNS6-GFP and rPDCoV-ΔNS7. (C) PDCoV-, rPDCoV-, rPDCoV-ΔNS6-GFP-, rPDCoV-ΔNS7- or mock-infected LLC-PK1 cells were detected by IFA using antibodies against PDCoV NS6, NS7, and N protein, respectively. GFP in rPDCoVs-infected cells was examined by fluorescence microscopy. (D) Western blot for PDCoV N, NS6, NS7, and NS7a. Protein was isolated from LLC-PK1 cells infected with PDCoV, rPDCoV, rPDCoV-ΔNS6-GFP, and rPDCoV-ΔNS7, respectively, which was harvested 24 hpi.

The availability of PDCoV full-length cDNA clones allowed us to investigate the role of PDCoV accessory proteins in viral replication. To this end, we constructed the NS6-deficient variant by replacing the NS6 gene in the PDCoV F fragment with that of the green fluorescent protein (GFP). Of note, the NS6 transcription regulatory sequence (TRS) was retained to regulate subgenomic RNA expression (Figure 2A). To construct the NS7 knockout virus, initiation codons ATG and the following seven downstream ATGs of the NS7 gene from nucleotides 24084 to 24629 in the PDCoV F fragment were changed to ACGs to completely abolish NS7 gene expression, but not altering the amino acid sequence of the PDCoV N protein by silent mutations. In the NS7 deletion mutants, NS7a expression was also abolished because it shared the same ORF with NS7 and the start ATG codon of NS7a (nucleotides 24384–24386) had been changed to ACG in the rPDCoV-ΔNS7 cDNA clone.

Following electroporation of the full-length transcripts into LLC-PK1 cells, rPDCoV-ΔNS6-GFP and rPDCoV-ΔNS7 were rescued, separately, and subject to three rounds of plaque purification. Sequence analyses of the recombinant viruses’ full-length genomes revealed that the NS6 gene was successfully replaced by GFP in rPDCoV-ΔNS6-GFP and the NS7 gene was disrupted by several nucleotide substitutions in rPDCoV-ΔNS7 (Figure 2B). No unwanted nucleotide change was found in their genomes compared to their cDNA clones. Furthermore, the infectivity of mutant viruses in LLC-PK1 cells was confirmed by IFA staining using antibodies specific for the N, NS6, and NS7 protein, respectively. As expected, NS6 or NS7 expression was not detected in cells infected with mutants with disrupted NS6 or NS7, whereas N expression was apparent in those infected by all recombinant viruses (Figure 2C). Strong GFP signal was also detected in cells infected with rPDCoV-ΔNS6-GFP (Figure 2C), suggesting the viral sgmRNA corresponding to the GFP gene was expressed effectively. In addition, the NS6, NS7 and N proteins expression in the cell culture infected by various recombinant viruses were also examined by western blot analysis (Figure 2D), which were in congruence with results of the IFA. It is noteworthy that the expression of NS7a was also abolished in the rPDCoV-ΔNS7-infected cells (Figure 2C) as its initiation codon was also altered (from ATG to ACG) in the rPDCoV-ΔNS7 cDNA clone. These data collectively suggested that recombinant viruses with NS6 and NS7 deletion were successfully rescued *in vitro*.

### Characterization of recombinant viruses

**Figure 3.**
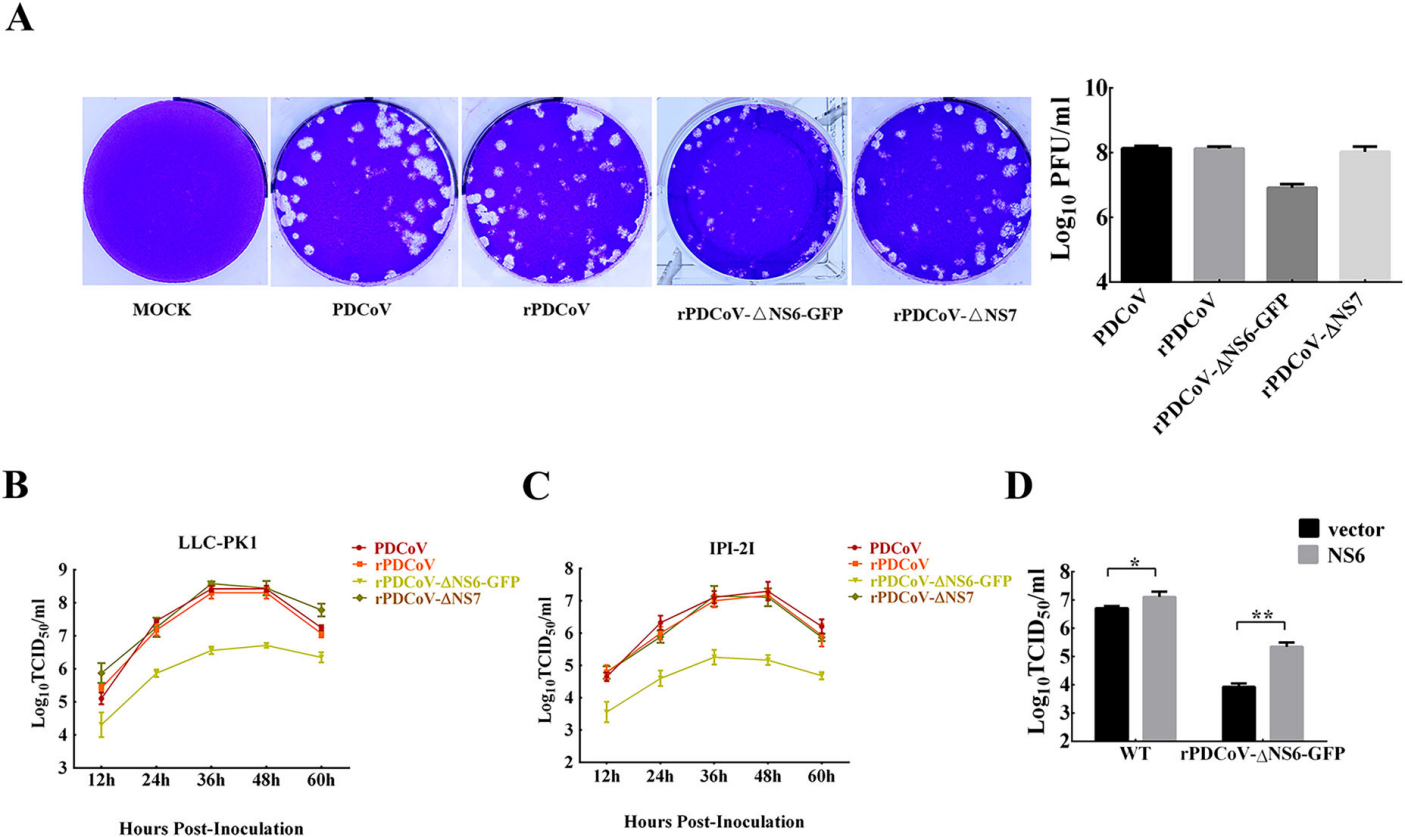
Plaque morphology and recombinant virus growth kinetics. (A) Representative plaques of mock-, PDCoV-, rPDCoV-, rPDCoV-ΔNS6-GFP- and rPDCoV-ΔNS7-infected LLC-PK1 cells at 2 dpi. (B, C) The growth curves of PDCoV, rPDCoV, rPDCoV-ΔNS6-GFP and rPDCoV-ΔNS7 in LLC-PK1 cells (B) and IPI-2I cells (C). Cells were infected with PDCoV, rPDCoV, rPDCoV-ΔNS6-GFP and rPDCoV-ΔNS7 at an MOI of 0.01, and cell cultures were collected at indicated times to determine the viral titers by TCID_50_. (D) Complementation assay of rPDCoV-ΔNS6-GFP infection in LLC-PK1 cells transiently expressing NS6. LLC-PK1 cells were transfected with the pCAGGS expressing NSP6 or empty vector, and were subsequently infected with PDCoV or rPDCoV-ΔNS6-GFP. Cell cultures were collected at 24 hpi for the TCID_50_ assay.

To evaluate the functional role of the accessory proteins NS6 and NS7 in PDCoV replication, we infected LLC-PK1 cells with each recombinant virus and determined the viral plaque size as well as the growth kinetics. As depicted in Figure 3A, rPDCoV and rPDCoV-ΔNS7 produced plaque morphologies similar to those of wild-type PDCoV, while rPDCoV-ΔNS6-GFP exhibited markedly reduced plaque size compared to PDCoV and rPDCoV. The growth kinetics of rPDCoV-ΔNS7 resembled those of PDCoV and rPDCoV, reaching peak virus titers around 10^9^ TCID_50_/ml at 36 h post-infection (Figure 3B). However, the viral titer of rPDCoV-ΔNS6-GFP in LLC-PK1 cells was approximately 10–70 fold lower relative to that of the wild-type virus (Figure 3B). A very similar growth pattern was also observed in infected IPI-2I cells (Figure 3C). To further explore whether the reduced viral yield of rPDCoV-ΔNS6-GFP was due to the absence of NS6 expression, we performed a complementation analysis in LLC-PK1 cells transiently expressing NS6 protein. Interestingly, cells transfected with the NS6-expressing plasmid exhibited significant enhancement of viral titers for both PDCoV and rPDCoV-ΔNS6-GFP, compared to those transfected with empty vector (Figure 3D). Taken together, these data indicated that the NS6 protein, but not NS7 protein, is critical for PDCoV replication *in vitro*.

### Virulence, inflammatory cytokines mRNA expressions and replication of recombinant viruses in newborn piglets

**Figure 4.**
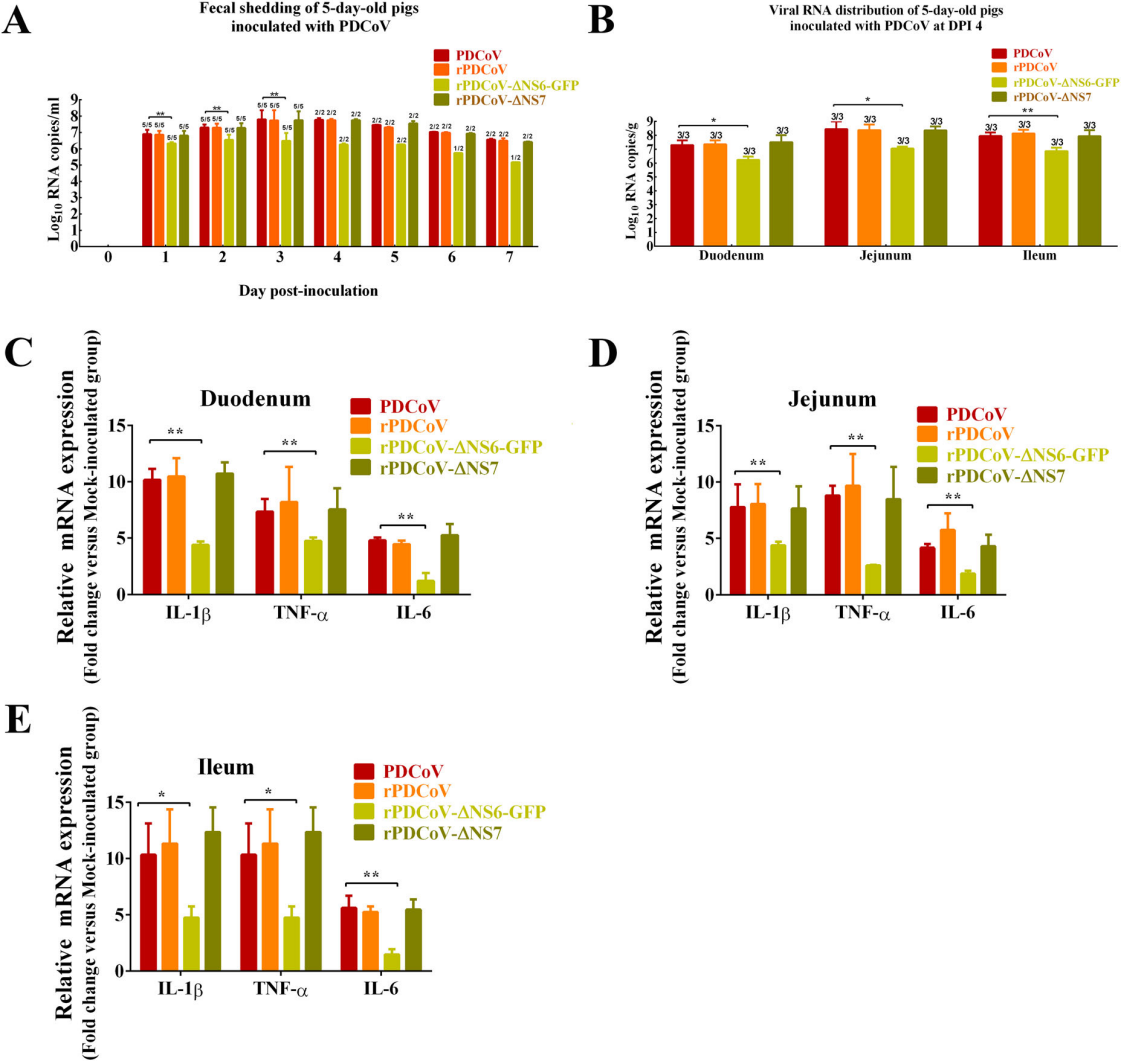
Fecal viral shedding, virus distribution and inflammatory cytokines expressions in PDCoV-, rPDCoV-, rPDCoV-ΔNS6-GFP- and rPDCoV-ΔNS7-inoculated piglets. (A) Fecal viral shedding in pigs orally fed with PDCoV, rPDCoV, rPDCoV-ΔNS6-GFP or rPDCoV-ΔNS7. (B) Virus distribution at 4 DPI in pigs inoculated with PDCoV, rPDCoV, rPDCoV-ΔNS6-GFP or rPDCoV-ΔNS7. (C, D, E) IL-1β, TNF-α, and IL-6 mRNA expressions in intestinal tissue of piglets inoculated with PDCoV, rPDCoV, rPDCoV-ΔNS6-GFP or rPDCoV-ΔNS7, respectively.

To determine the role of the accessory proteins in regulating viral growth and virulence *in vivo*, newborn piglets were orally inoculated with wild-type PDCoV, rPDCoV, rPDCoV-ΔNS6-GFP, or rPDCoV-ΔNS7, respectively. Within 2 days post-inoculation (DPI), all piglets orally fed with PDCoV-, rPDCoV-, and rPDCoV-ΔNS7 experienced varying degrees of diarrhea, vomiting, lethargy, and anorexia (Table 1). No significant differences in the clinical fecal scores were noted among rPDCoV-ΔNS7-, rPDCoV and PDCoV-inoculated groups (Table 1). Interestingly, piglets inoculated with rPDCoV-ΔNS6-GFP showed hardly detectable clinical symptoms throughout the course of the experiment. Of note, we could still detect the fecal PDCoV RNA levels in rPDCoV-ΔNS6-GFP-inoculated piglets at 1–7 DPI, but they were significantly lower compared to other groups (Figure 4A). To investigate the *in vivo* proliferative capacity and pathogenicity between PDCoV and its deletion mutants, three pigs from each group were randomly selected and sacrificed on 4 DPI. Although the differences in the viral RNA copy numbers detected in duodenums, jejunum and ileums were not significant among rPDCoV-ΔNS7-, rPDCoV- and PDCoV-inoculated groups, the viral RNA copy numbers were significantly decreased (10–20 fold) in intestinal tissues obtained from icPDCoV-ΔNS6-GFP-inoculated piglets (Figure 4B).

**Table 1.** Summary of PDCoV pathogenesis in 5-day-old piglets^a^.

Group	No. of pigs	Pig	Highest fecal score^b^	Onset of diarrhea (dpi)	Age (dpi) of piglet at Lethargy & anorexia (day)	Jejunum VH:CD, Mean (±SD)	Antigen detection in the intestine^c^		
							Duodenum	Jejunum	Ileum
MOCK	5	1	1	NA	NA	5.46 (0.7)	−		−
		2	1	NA	NA	ND	ND	ND	ND
		3	1	NA	NA	ND	ND	ND	ND
		4	1	NA	NA	5.17 (0.4)	−	−	−
		5	1	NA	NA	5.40 (0.7)	−	−	−
PDCoV	5	6	3	1	6 (1)	ND	ND	ND	ND
		7	3	1	6 (1)	1.66 (0.4)	+	++	+++
		8	3	2	7 (2)	1.54 (0.4)	−	++	+++
		9	3	2	7 (2)	ND	ND	ND	ND
		10	3	1	6 (1)	1.97 (0.5)	+	++	+++
rPDCoV	5	11	3	2	7 (2)	ND	ND	ND	ND
		12	3	1	6 (1)	ND	ND	ND	ND
		13	3	2	7 (2)	1.80 (0.2)	+	++	+++
		14	3	1	6 (1)	1.96 (0.3)	+	++	+++
		15	3	1	6 (1)	1.62 (0.4)	+	++	+++
rPDCoV-ΔNS6-GFP	5	16	2	NA	NA	5.39 (0.6)	−	+	+
		17	1	NA	NA	5.25 (0.7)	−	+	+
		18	1	NA	NA	5.28 (0.5)	−	−	+
		19	1	NA	NA	ND	ND	ND	ND
		20	1	NA	NA	ND	ND	ND	ND
rPDCoV-ΔNS7	5	21	3	1	6 (1)	ND	ND	ND	ND
		22	3	2	7 (2)	1.92 (0.5)	+	++	+++
		23	3	1	6 (1)	1.70 (0.3)	−	++	+++
		24	3	1	6 (1)	1.71 (0.2)	+	++	+++
		25	3	2	7 (2)	ND	ND	ND	ND

^a^NA, not available; ND, not done because piglets were not for necropsy; VH:CD, villous height/crypt depth.

^b^Fecal scores were as follows: 0, normal; 1, pasty stool; 2, semiliquid diarrhea; and 3, liquid diarrhea.

^c^Antigen detection by immunohistochemical staining: −, no positive cells showed a positive signal; +, 1%–30% of villous enterocytes showed a positive signal; ++, 31–60% of villous enterocytes showed a positive signal; +++, 61%–100% of villous enterocytes showed a positive signal.

The inflammatory cytokines mRNA expressions in intestinal tissue were also detected by RT-qPCR. As shown in the Figure 4C–E, proinflammatory cytokines such as IL-1β, TNF-α, and IL-6 showed increased levels in the duodenum, jejunum and ileum of piglets inoculated with PDCoV, rPDCoV, rPDCoV-ΔNS6-GFP and rPDCoV-ΔNS7 compared to mock-inoculated group. Moreover, the mRNA levels of IL-1β, TNF-α and IL-6 in the intestinal tissues obtained from rPDCoV-ΔNS6-GFP-infected piglets were significantly decreased compared to the wild-type PDCoV group, while there were no significant differences in IL-1β, TNF-α and IL-6 expression detected in duodenums, jejunum and ileums among rPDCoV-ΔNS7-, rPDCoV- and PDCoV-inoculated groups (Figure 4C–E).

**Figure 5.**
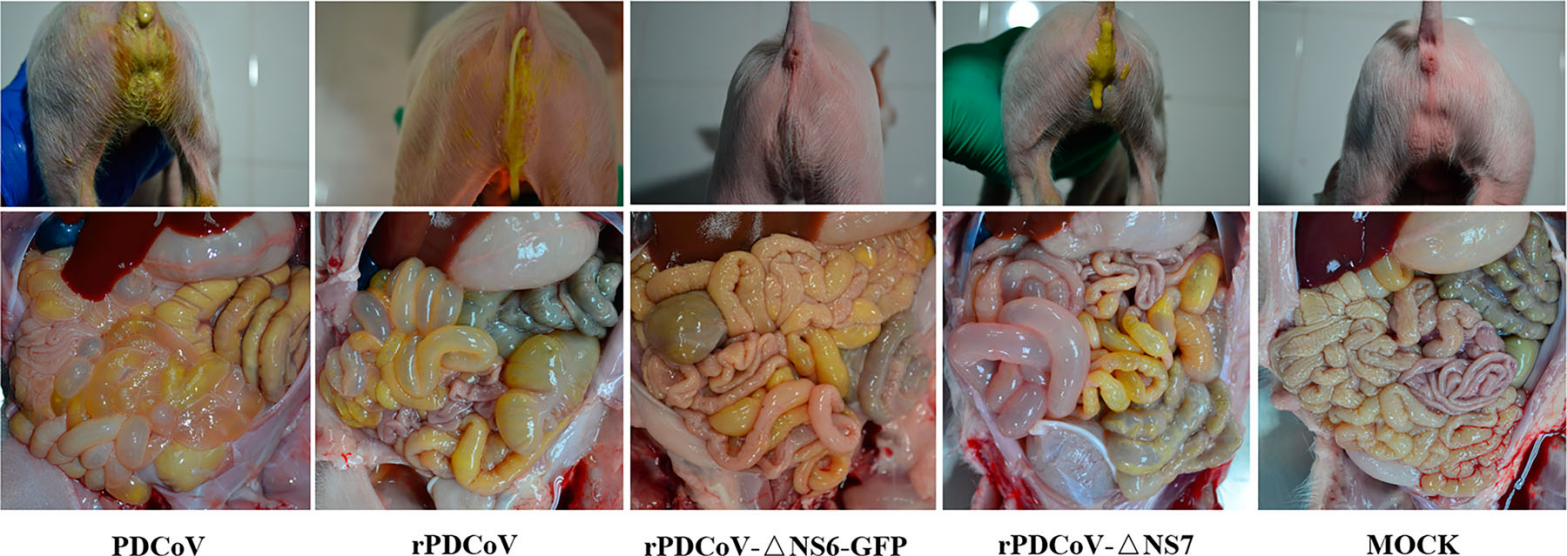
Clinical assessment of piglets challenged with PDCoV, rPDCoV, rPDCoV-ΔNS6-GFP or rPDCoV-ΔNS7. Five-day-old pigs at 4 DPI with PDCoV, rPDCoV, rPDCoV-ΔNS6-GFP, rPDCoV-ΔNS7 or DMEM medium.

**Figure 6.**
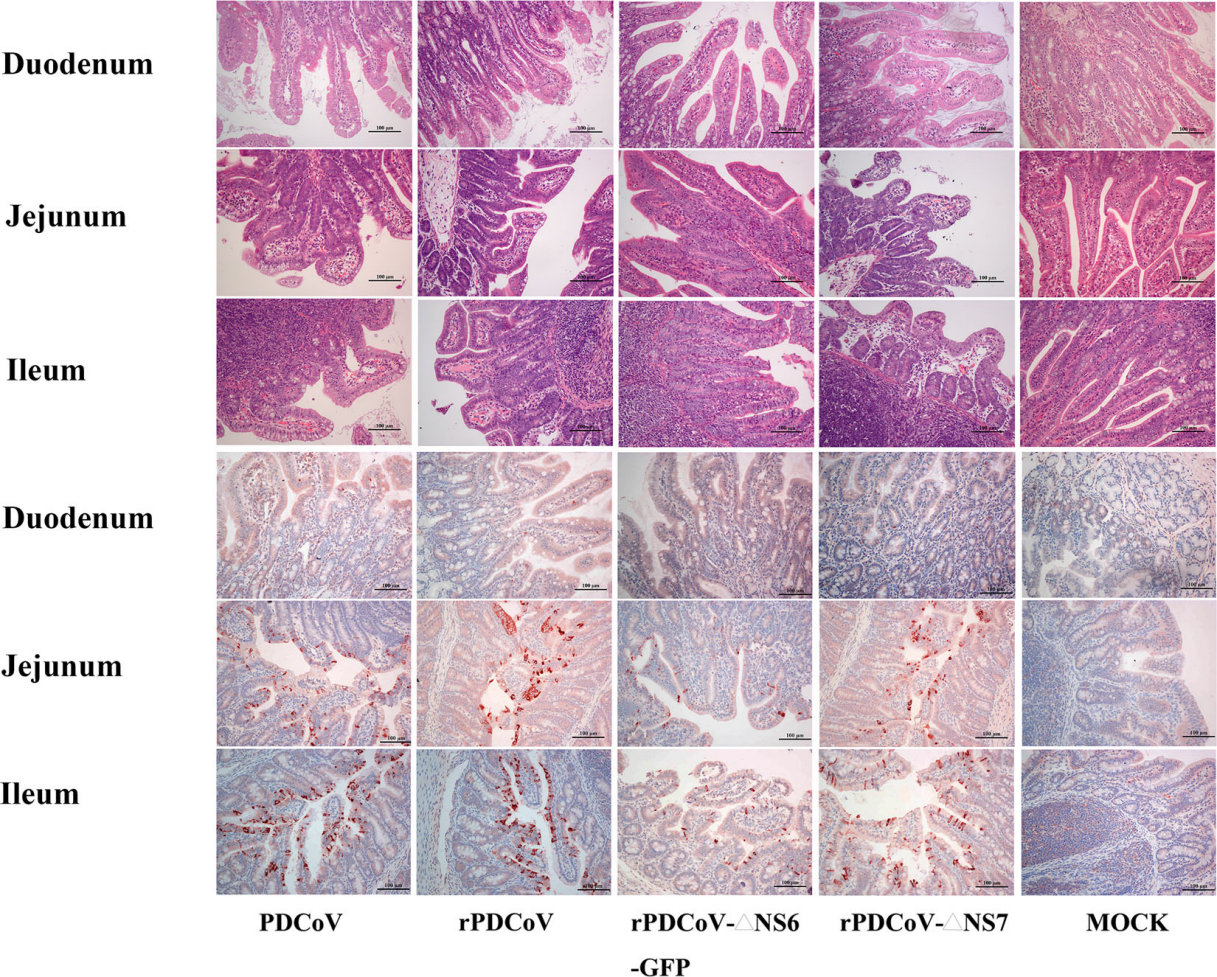
Histology and IHC staining of rPDCoV-infected piglet intestine. HE and IHC staining of the duodenum, jejunum and ileum of piglets inoculated with PDCoV, rPDCoV, rPDCoV-ΔNS6-GFP or rPDCoV-ΔNS7. Scale bars are shown in each picture.

Necropsy examinations showed the transparent small intestines with thin-walled, and gas-distended in the rPDCoV-ΔNS7-, rPDCoV- and PDCoV-inoculated groups (Figure 5). In striking contrast, small intestines appeared grossly normal in rPDCoV-ΔNS6-GFP-infected piglets, similar to those of the mock-infected group (Figure 5). Moreover, microscopic lesions of the jejunum and ileum obtained from rPDCoV-ΔNS7-, rPDCoV- and PDCoV-inoculated piglets were apparently observed, while no significant microscopic lesions were found in any of these sections from rPDCoV-ΔNS6-GFP-inoculated piglets. Notably, severe necrosis, vacuolation and villous atrophy of the small intestinal enterocytes were observed in rPDCoV-ΔNS7-, rPDCoV- and PDCoV-inoculated piglets rather than rPDCoV-ΔNS6-GFP-piglets (Figure 6). The ratios of villus height to crypt depth (VH/CD) of the jejunum were significantly higher in mock- or rPDCoV-ΔNS6-GFP-inoculated piglets than those of rPDCoV- and PDCoV-inoculated piglets (Table 1). However, the VH/CD ratios of the jejunum of rPDCoV-ΔNS7-inoculated group was similar to those of rPDCoV- and PDCoV-inoculated piglets (Table 1). Immunohistochemistry (IHC) staining with anti-PDCoV N specific antibodies suggested the presence of parental and recombinant viruses in all segments of small intestines, especially in jejunum, and ileum (Figure 6; Table 1). Notably, the PDCoV antigens in the villous enterocytes were only marginally expressed in rPDCoV-ΔNS6-GFP-infected piglets, in line with the results in viral RNA detection (Figure 4B).

Collectively, these results demonstrated that the disruption of NS6 in PDCoV greatly impaired the viral replication, resulting in significant attenuation of the virus in piglets. The lack of NS7, however, has a minimal effect on viral replication and virulence *in vivo*.

## Discussion

Emerging coronaviruses cause a wide range of diseases in humans and animals, posing a significant threat to human health or resulting in enormous economic losses to the global livestock and trade industries. Until now, at least six pathogenic coronaviruses have been reported in swine, including porcine epidemic diarrhea virus (PEDV), transmissible gastroenteritis virus (TGEV), Swine Acute Diarrhea Syndrome Coronavirus (SADS-CoV), porcine respiratory coronavirus (PRCV), porcine hemagglutinating encephalomyelitis virus (PHEV) and Porcine deltacoronavirus (PDCoV). PDCoV is among newly emerged enteropathogenic coronaviruses known to cause severe diarrhea in suckling piglets. To date, no effective vaccines are available to control PDCoV outbreaks. In this study, we successfully constructed the full-length cDNA clone of PDCoV strain CHN-HG-2017 to rescue the infectious PDCoV *in vitro*, the first reverse genetic system for deltacoronaviruses until now. This technology platform is currently needed for the rational design of attenuated viruses. In addition, this infectious clone serves as a useful molecular tool for systematically investigating the role of viral genes in deltacoronavirus replication, pathogenesis, and virulence.

The full-length cDNA clone of PDCoV was assembled from a panel of six adjoining fragments using *in vitro* ligation; an approach successfully utilized for developing the infectious clones of TGEV, MHV, IBV, NL63, SARS-CoV, MERS-CoV, and PEDV [27,31–36]. The full-length sequence of rPDCoV was identical to the parental PDCoV strain, except for three silent mutations introduced to remove BsmBI restriction sites. The parental PDCoV and rPDCoV demonstrated similar biological characteristics. Both caused indistinguishable CPE in LLC-PK1 cells (Figure 1B), exhibited comparable levels of protein expression (Figure 2D), generated nearly alike plaque size in LLC-PK1 cells (Figure 3A), and replicated with similar growth kinetics in LLC-PK1 and IPI-2I cells (Figure 3B and C).

Like other delta-CoVs, the PDCoV encodes two specific accessory genes namely NS6, located between the M and N genes, and NS7, occupied within the N gene [37]. Currently, the role of accessory proteins in the replication of delta-CoVs are not clearly elucidated. Here, we employed the reverse genetics to construct recombinant PDCoVs with abortive NS6 or NS7 genes and demonstrated that both NS6 and NS7 were not essential for PDCoV replication *in vitro* (Figure 2C and D). In addition, we showed, using rPDCoV-ΔNS7, that the NS7a, a newly identified accessory protein encoded by PDCoV [30], is also not required for efficient replication in LLC-PK1 cells. However, it is important to emphasize that while rPDCoV-ΔNS7 with both NS7 and NS7a deletion exhibited similar growth kinetics with the parental virus *in vitro*, rPDCoV lacking the functional NS6 displayed markedly lower replication efficiency (Figure 3B and C). These data thus suggested that PDCoV accessory protein NS6, but not NS7, is very like play a key role in promoting viral replication.

Although dispensable for viral replication, coronavirus accessory proteins usually play pivotal roles in viral infection and pathogenesis *in vivo*. Menachery et al. reported that deletion of ORF3, ORF4a, ORF4b, and ORF5 in MERS-CoV led to a significant attenuation of the virus in mice [38]. On the other hand, newborn piglets infected with TGEV lacking protein 7 expression developed a faster and more pronounced clinical disease compared with the parental virus [39]. In this study, piglets infected with rPDCoV-ΔNS6-GFP showed minimal clinical signs, with significantly lower viral RNA copy numbers in small intestines compared with wild-type virus. In contrast, piglets orally fed with rPDCoV-ΔNS7 exhibited clinical manifestations and pathological injury similar to those infected with the wild-type virus (Figures 4–6, Table 1). These results suggested that the NS6 protein is necessary for efficient viral replication *in vivo*, which may be attributable to the high pathogenicity of PDCoV. Recently, Fang et al. demonstrated that the PDCoV NS6 predominantly localize to the ER complex and ER-Golgi intermediate compartment, which is the site of coronavirus assembly and packaging [29], suggesting that NS6 may be involved in virus assembly. Moreover, PDCoV NS6 was identified as an inhibitor of IFN-β expression by interacting with RIG-I and MDA5 to impede their association with double-stranded RNA, thus disturbing RLR-mediated IFN-β signal pathway [40]. It is thus probable that the NS6 protein might contribute to PDCoV pathogenesis by, at least in part, regulating virus-host interaction. Given that rPDCoV-ΔNS6-GFP is highly attenuated *in vivo*, it could be considered a promising vaccine candidate for PDCoV. Although immunological profiles of pigs inoculated with rPDCoV-ΔNS6-GFP are still needed to fully support its applicability, our study here provides the basis for rational development of recombinant live attenuated PDCoV vaccines.

In summary, we demonstrate for the first time a reverse genetics platform of PDCoV, which has allowed the future identification of viral genes involved in replication and pathogenesis. In addition, we engineered a recombinant PDCoV to efficiently express GFP, which could be applied as a tool for high-throughput drug screens and neutralizing antibody assays. Furthermore, deletion of the accessory gene NS6 in PDCoV genome resulted in an attenuated viral phenotype, thereby providing an attractive strategy to generate live attenuated PDCoV vaccines.

## Materials and methods

### Cells and viruses

LLC-PK1 cells were obtained from the American Type Culture Collection (ATCC CL-101) and cultured in Dulbecco’s modified Eagle’s medium (DMEM) supplemented with 10% fetal bovine serum (FBS, Gibco). Porcine ileum-derived IPI-2I cells were kindly provided by Lisheng Zhang (Huazhong Agricultural University, China) and were grown in RPMI 1640 medium with 10% FBS (Gibco). The wild-type CHN-HG-2017 strain of PDCoV was cultured on LLC-PK1 cells. The virus was grown in maintenance medium containing DMEM with 10% tryptose phosphate broth (TPB) and 10 μg/ml trypsin (Sigma). Virus titrations were performed on LLC-PK1 cells following standard procedures by 50% cell culture infectious dose (TCID_50_) or plaque assays, as described previously [41]. For TCID_50_ assays, CPE was recorded at 48 h postinfection (h.p.i.). For plaque assays, cells were fixed with 10% formaldehyde and stained with crystal violet.

### Assembly of a full-length cDNA clone of PDCoV

The cloning strategy for a full-length PDCoV cDNA clone is illustrated in Figure 1A, using six contiguous fragments flanked by the BsmBI restriction endonuclease sites that leave nonpalindromic overhangs. All cDNA fragments were synthesized based on PDCoV passage 3 sequence (GenBank accession number MF095123.1) and were ligated in the pJET1.2/blunt Cloning Vector (Thermo Scientific). Three naturally occurring BsmBI restriction sites were removed by silent mutations to produce unique BsmBI sites at appropriate locations across the PDCoV genome. Sequences of all PDCoV fragments were verified by sequencing after plasmids preparation from bacterial cultures. The plasmids containing PDCoV fragments were digested or double-digested with BsmBI, MluI, or XhoI designated in Figure 1A, separated on a 0.8% agarose gel, excised, and purified using a QIAquick gel extraction kit (Qiagen). The digested PDCoV fragments were mixed and ligated overnight at 4°C using T4 DNA ligase (Thermo Scientific), phenol/chloroform extracted, and precipitated with isopropyl alcohol. Full-length RNA transcripts capped with m^7^G(5′)ppp(5′)G were generated using mMESSAGE mMACHINE T7 transcription kit according to the manufacturer’s instructions (combine 5 μL PDCoV ligated DNA, 15 μL 2× NTP/CAP, 4 μL GTP, 3 μL 10× T7 buffer, and 3 μL T7 enzyme). In addition, SP6 PDCoV N gene transcripts were generated from the PCR-purified PDCoV N gene sample using a 4:1 ratio of cap to GTP (Ambion).

### Construction of PDCoV cDNA clones lacking NS6 or NS7 genes

To replace the NS6 with the GFP construct, the GFP gene was amplified by PCR with flanking PDCoV sequence using primers: GFP-F, 5′-ctgtataagtatatgtaATGGTGAGCAAGGGCGAG-3′ and GFP-R, 5′-tagattggtgtcaaaactTTACTTGTACAGCTCGTC-3′. The entire plasmid containing PDCoV-F except for the region of NS6 gene was amplified by PCR with primers: PDCoV-F-ΔNS6-F, 5′-agttttgacaccaatctatcatgg-3′ and PDCoV-F-ΔNS6-R, 5′-ttacatatacttatacaggcgagc-3′. The two fragments were purified, mixed and connected using the In-Fusion HD Cloning Kit (TAKARA). PDCoV-F-ΔNS6-GFP was cultured and sequenced to ensure the replacement of NS6 with GFP containing the NS6 TRS and no mutation in other regions of PDCoV-F.

To generate the NS7 deletion construct, the initiation codons ATG and the following seven downstream ATGs of the NS7 gene in the PDCoV F fragment were changed to ACGs to block NS7 gene expression (Figure 2A). The mutant NS7 gene was synthesized and replaced the original NS7 gene in the plasmid containing PDCoV-F using In-Fusion HD Cloning Plus kits (TAKARA). PDCoV-F-ΔNS7 was cultured in *E. coli* DH10B cells and sequenced to ensure the modified NS7 gene containing the desired mutations.

### In vitro transfection

The 30 µl of full-length PDCoV RNA and 10 µl of N RNA transcripts were mixed and added to 800 µl of LLC-PK1 cells (8.0 × 10^6^ cells/ml) in phosphate buffered saline (PBS). Three pulses of 450 V at 50 µF were given with the Gene Pulser Xcell electroporation system (Bio-Rad). The transfected cells were seeded to a 75-cm^2^ flask in growth medium at 37°C overnight, after which time the cells were washed with PBS and incubated in maintenance medium. Rescued viruses were then purified by plaque assay.

### Virus genome sequencing

Viral RNA was extracted from LLC-PK1 cells infected by wild-type PDCoV or each rescued rPDCoVs using TRIzol reagent (Invitrogen, USA) according to the manufacturer’s instructions. cDNA was synthesized from extracted RNA with specific oligonucleotides using a PrimeScript RT reagent kit (Takara, Japan). The whole genome of wild-type PDCoV and each rescued rPDCoVs was sequenced by RT–PCR and 5′/3′ RACE as previously described [5]. Genome sequence assembly and alignment with the consensus sequence of the CHN-HG-2017 strain were performed using the SeqMan and MegAlign programs of the LASERGENE bioinformatics computing suite (DNASTAR, Madison, WI).

### Antibodies

Polyclonal antibodies (pAbs) specific for PDCoV NS6 and NS7 proteins were prepared for the detection of their expression in PDCoV- or rPDCoVs-infected LLC-PK1 cells. In brief, the full-length PDCoV NS6 and truncated PDCoV NS7 (amino acids 1–160) cDNA were subcloned into pET28a vector and expressed in *Escherichia coli* Rosetta (DE3) under IPTG induction. Subsequently, female BALB/c mice were immunized with the purified recombinant NS6 or NS7 proteins. Their sera were collected, and polyclonal antibodies against NS6 and NS7 were purified and verified by Western-blot and immunofluorescence analysis, respectively. Anti-PDCoV N protein mAb was generated as previously described [5].

### Indirect immunofluorescence assay

LLC-PK1 cells were seeded on sterilized coverslips placed in 24-well plates. At 24 h.p.i, the cells were fixed in 4% paraformaldehyde for 15 min, permeabilized in PBS containing 0.1% Triton X-100 for 10 min. Subsequently, cells were blocked with 5% skimmed milk in PBS and incubated with anti-N protein mAb, anti-NS6 or anti-NS7 pAb, respectively. Following treatment of AlexaFluor-594 (red)-labeled donkey anti-mouse IgG secondary antibodies (Invitrogen), cells were stained with 4′,6-diamidino-2-phenylindole (Invitrogen) for 15 min at room temperature. Fluorescence was examined using a Zeiss LSM410 system.

### Western blot

Virus-infected LLC-PK1 cells were harvested with lysis buffer (65 mM Tris-HCl [pH 6.8], 4% sodium dodecyl sulfate, 3% DL-dithiothreitol and 40% glycerol). Proteins isolated from the lysate were separated by 12% SDS-PAGE and then transferred to a polyvinylidene difluoride membrane. The membrane was incubated with antibodies against N protein, NS6, and NS7, respectively, after blocking with 10% dry milk. After washing in PBST, the membrane was incubated with horseradish peroxidase (HRP)-conjugated goat anti-mouse IgG (ABclonal). Signals were detected using the SuperSignal West Pico Luminal kit (Pierce).

### Plasmid transfection

PDCoV NS6 cDNA was subcloned into the pCAGGS vector with a N-terminal HA tag for expression. LLC-PK1 cells were seeded in the 24-well plates and grown to approximately 80% confluence. Cells were transfected with 1 µg/well with indicated expression plasmids or an empty vector. Transfection was carried out with Lipofectamine 2000 reagent (Invitrogen) according to the manufacturer’s instructions.

### Animal studies

The animal study protocol was reviewed and approved by the Scientific Ethic Committee of Huazhong Agricultural University (HZAUSW-2018-006). Twenty-five 5-day-old piglets were purchased from a commercial pig farm and were randomly divided into five groups housed in separated containments (Table 1). All piglets were confirmed negative for PDCoV, PEDV, TGEV, and rotavirus by virus-specific RT-PCRs on rectal swabs. Neutralizing antibodies against PDCoV were not detected in sera of the piglets or sow. After 1 day acclimation, piglets were orally challenged with 10 ml of PDCoV, rPDCoV, rPDCoV -ΔNS6-GFP, or rPDCoV-ΔNS7 at a dose of 1.0 × 10^7^ TCID_50_, while the mock-infected group was inoculated with 10 ml of maintenance medium. Piglets were monitored daily for the presence of vomiting and clinical signs of diarrhea, lethargy, and anorexia. Diarrhea severity was scored with the following criteria: 0, normal; 1, pasty stool; 2, semiliquid diarrhea; and 3, liquid diarrhea. Rectal swabs were collected daily for detecting virus shedding by a quantitative PDCoV N-gene based real-time RT-PCR. At 4 DPI, three piglets in each group were randomly selected and necropsied, the different sections of the small intestine (duodenum, jejunum, ileum) were collected for detection of viral RNA by RT-qPCR and histopathological examination by hematoxylin and eosin (H&E) staining. Rectal swabs and tissue samples collection, RT-qPCR testing, and histopathology were performed as previously described [5]. To detect the inflammatory cytokines mRNA expressions in intestinal tissues, RT-qPCR was performed on the Applied Biosystem 7500 Fast Real-time PCR System (Life Technologies, USA) using FastStart Universal SYBR^@^ Green Master Mix (Roche, Germany) with specific primers in Table 2. And the conditions as follows: 95°C for 10 min, and 40 cycles of 95°C for 15 s, 60°C for 30 s. Data were normalized to GAPDH expression and are expressed as fold differences using the 2^−ΔΔCT^ method.

**Table 2.** Primers used for RT-PCR.

Target gene	Primer sequence (5′ to 3′)
GAPDH	F ^a^: ATGGTGAAGGTCGGAGTGAAC
	R ^b^: GGCGACAATGTCCACTTTGC
IL-1β	F: AGCATGCCAATGGTTTTCTCT
	R: CCAGCACCAGGGCTTTTTC
IL-6	F: CCTCGGCAAAATCTCTGCAA
	R: TGAAACTCCACAAGACCGGT
TNF-α	F: TCCACCAACGTTTTCCTCACT
	R: AGGGCTCTTGATGGCAGAGA

^a^Forward primer; ^b^Reverse primer.

### IHC

Serial sections of duodenum, jejunum, ileum at 4 DPI necropsy were evaluated for PDCoV antigen by IHC using a PDCoV-N-specific mAb as previously described. The IHC signal of PDCoV antigen was semi-quantitatively scored with the following criteria: −, no positive cells showed a positive signal; +, 1%–30% of villous enterocytes showed a positive signal; ++, 31–60% of villous enterocytes showed a positive signal; +++, 61%–100% of villous enterocytes showed a positive signal.
